# Low frequency of c-MPL gene mutations in Iranian patients with Philadelphia-negative myeloproliferative disorders

**Published:** 2015-03-15

**Authors:** A Ghotaslou, F Nadali, B Chahardouli, N Alizad Ghandforosh, SH Rostami, K Alimoghaddam, A Ghavamzadeh

**Affiliations:** 1 MS.c Student , Department of Hematology,School of Allied Medical Sciences , Tehran university of Medical Sciences, Tehran , Iran; 2 Associate Professor, Departement of Hematology, School of Allied Medical Sciences , Tehran university of Medical Sciences , Tehran, Iran; 3 Assistant Professor, Hematology-Oncology and Stem cell Transplantation Research Center, Tehran university of Medical Sciences, Tehran, Iran; 4 MS.c Student, Hematology-Oncology and Stem cell Transplantation Research Center, Tehran university of Medical Sciences, Tehran, Iran; 5 Professor, Hematology-Oncology and Stem cell Transplantation Research Center, Tehran university of Medical Sciences, Tehran, Iran

**Keywords:** ARMS –PCR, c- MPL mutation, JAK2V617F, Myeloproliferative Disorders

## Abstract

**Background:**

Myeloproliferative disorders are a group of diseases characterized by increased proliferation of myeloid lineage. In addition to JAK2V617F mutation, several mutations in the c-MPL gene have been reported in patients with philadelphia-negative chronic myeloproliferative disorders that could be important in the pathogenesis of diseases. The aim of the present study was to investigate the frequency of c-MPL and JAK2V617F mutations in Iranian patients with Philadelphia-negativemyeloproliferative disorders.

**Material and Methods:**

Peripheral blood samples were collected from 60 patients with Philadelphia-negative MPD) Subgroups ET and PMF) and 25 healthy subjects as control group. The mutation status of c-MPL and Jak2V617F were investigated by using Amplification-refractory mutation system (ARMS) and Allele-Specific PCR (AS-PCR), respectively. The results were confirmed by sequencing.

**Results:**

Among 60 patients, 34 (56.6%) and 1(1.7%) had Jak2V617F and c-MPL mutation, respectively. Patients with Jak2V617F mutation had higher WBC counts and hemoglobin concentration than those without the mutation (p= 0.005, p=0.003). In addition, for all healthy subjects in control group, mutations were negative.

**Conclusions:**

The present study revealed that the c-MPL mutations unlike the Jak2V617F mutations are rare in Iranian patients with Ph-negative MPNs and the low mutation rate should be considered in the design of screening strategies of MPD patients.

## Introduction

Myeloproliferative Disorders (MPD) or Myeloproliferative neoplasms (MPN) recognized as a group of clonal diseases of hematopoietic stem cells which identified by increasing proliferation of myeloid lineage with a prolonged and sometimes indolent clinical course. Polycythemia vera (PV), Essential thrombocythemia(ET), and Primary Myelofibrosis (PMF) are three main members of the BCR-ABL negative MPNs. They are associated with thrombosis , hemorrhage, splenomegaly, and risk of conversion to acute myeloid leukemia.[1-3] Diagnostic criteria for PV , ET and PMF, which have been accepted by the WHO, includes determination of clonality and also investigation of JAK2V617F mutation . JAK2V617F mutation was found in most patients with PV  and about half of the cases with ET and PMF[4, 5]. The mutation is characterized by a guanine to thymine substitution at nucleotide 1849 in exon 12 of the JAK2 gene that causes a valine to phenylalanine substitution at codon 617.This mutation in the absence of cytokine leads to the activation of JAK2. So, JAK2V617F mutation is considered like a predisposing factor for MPNs progress. Results of different studies have shown that detection of JAK2V617F mutation not only has important role in diagnosis, but also the treatment of MPNs may be improve by JAK- STAT pathway inhibitors [6]. But A significant proportion of patients with ET and PMF are JAK2V617F negative.  Sequencing of thrombopoietin receptor (MPL) leading to diagnosis of several substitution mutations in ET and PMF patients. This mutation through the activation of JAK2-STAT transcription factors leads to cell proliferation. Binding of TPO to cellular domain of c-MPL causes dimerization of intracellular domain of receptor so that it causes JAK2 cross- phosphorylation. The phosphorylated and activated JAK2 can phosphorylate tyrosine in cytoplasmic domain of c-MPL and it provides a docking site for downstream signaling molecules .Studies from Western countries have shown that the approximate frequency of c-MPL gene mutations is 5-10% in PMF and ET patients [7-9]. Since detecting these mutations is  valuable in diagnosis and no study has been done to determine the frequency of this mutation in Iranian patients, this study planned to evaluate primarily the prevalence of c-MPL gene mutation in MPD patients (ET and PMF subgroups) and secondly, the relation of these mutations with laboratory and clinical results of patients. Because some patients may have both mutations (c-MPL and JAK2V617F), c-MPL mutations also investigated in patients with JAK2V617F mutation. Previous study of PV patients did not identify any c-MPL mutations; therefore, in this study we excluded patients with PV. 

## Materials and methods

**Samples, DNA and RNA extraction:**

In this present study, 60 JAK2 (V617F)-negative Ph-negative ET and PMF patients as well as 25 healthy individual as control, investigated for mutations in c-MPL exone10 and JAK2V617F. Each patient’s Clinical and laboratory data included WBC count , Hemoglobin concentration , platelet count, age, status of spleen ,and other information were extracted from his/her medical records. Informed consent obtained from all the subjects. 10ml of peripheral blood samples were collected in tubes containing EDTA anticoagulant. Genomic DNA was extracted by the salting-out method, and Total RNA was isolated using the TRIZOL reagent (Invitrogen, Carlsbad, CA), and cDNA was synthesized according to the manufacturer's instructions by using random hexamer(Fermentas).

**AS-PCR, ARMS-PCR and direct sequencing:**

**Figure1:**
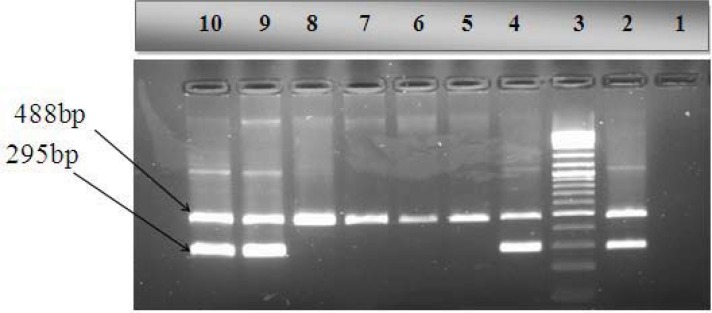
AS-PCR for detection of Jak2V617F mutation, from right to left lane 1 negative control, lane 2 positive control, lane 3 is 100–base pair (bp) marker. Lanes 4,9,10 are patients with mutation and lane 8 is patient without mutation and lanes 5, 6, 7 show healthy control.

**Figure2.**
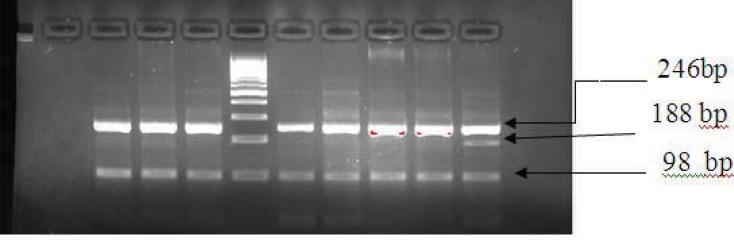
ARMS-PCR for detection of c-MPL mutation , from left to right lane 1 negative PCR control, lanes 2,3,4 healthy control , lane 5, 100–base pair (bp) DNA molecular weight marker, lanes 6,7,8,9 patients without mutation and lane 10 patient with c-MPL mutation . ARMS-PCR shows a 246bp control band, the 98bp band indicates the presence of the wild type allele and the 188bp band indicates the presence of the W515 mutation.

In order to investigate of JAK2V617F mutation AS-PCR technique and for c-MPL gene mutations, polymerase chain reaction-amplification refractory mutation system (PCR-ARMS) and direct DNA sequencing techniques were used. Primer sequences and PCR protocol have been previously described [10, 11]. Briefly, the 488bp and 299bp bands correspond to the wild-type and the mutant alleles, respectively in AS-PCR method (Figure1).To investigate of c-MPL gene mutations direct sequencing was conducted on DNA fragments amplified by the polymerase chain reaction,using primers flanking the MPL W515 mutation site. If a mutation was identified by direct sequencing, the sample would be used for ARMS PCR setup. In ARMS-PCR Outer primers generated 246bp (act as control), the 98bp band indicated the presence of the wild type allele and the 188bp band indicated the presence of the W515 mutation (Figure 2). PCR reaction’s products were evaluated on agarose gel 3%.

**Statistical analysis**

 The analysis was performed using SPSS Version 16 (spss INC, version16 IL, Chicago). The frequency of the mutations was explored for correlation with clinical and laboratory characteristics. The Chi square (χ2) test was used to compare baseline clinical characteristics of the patients with mutation status. A variable was considered significant when p < 0.05. 

## Results

Significant association was found between diseases subgroups (ET or PMF) and presence of JAK2V617F mutation (P=0.002). Compared to patients without the mutation, the median of WBC count and the mean of hemoglobin concentration in patients with JAK2 V617F mutation were higher (P=0.003 and P=0.005 respectively). Patients with JAK2V617F mutation also had a higher average platelet count (P=0.03) which seemed statistically significant, however, according to the correlation coefficient values, the changes are insignificant. On the other hand, based on the correlation coefficient 64.2 between patients subgroup and platelet count, this possibility was rejected and in fact, the cause of significance between the platelet count and JAK2V617F mutation could be due to asymmetric distribution of patients in ET group. The median platelet count in 31 ET patients , who had mutation, were ( 263.12 ± 945/5 *103/µl , P = 0.5) and the median platelet count in 3 PMF patients that had mutation were ( 382.32 ± 363/5, p = 0.9) .Of total 34 patients with JAK2V617F mutation, 20 cases (58.5%) had splenomegaly . No significant correlation was found between presence of mutation and splenomegaly (P=0.3) (Table 1). The only patient with c-MPL mutation was from ET subgroup, negative for JAK2 V617F mutation, male, 60 years old with platelet count 897 *103/µl and an enlarged spleen at the time of diagnosis. (Table 2) In bone marrow biopsy, moderate increase in the number of megakaryocyte without any symptoms of fibrosis was also seen. In this patient, sequence of c-MPL gene revealed that the mutation was due to replacing T with A nucleotide and patient’s mutation was W515R(Table 2 ). Moreover, c-MPL gene mutation (W515) was not found among the 25 studied healthy individual.

**Figure3:**
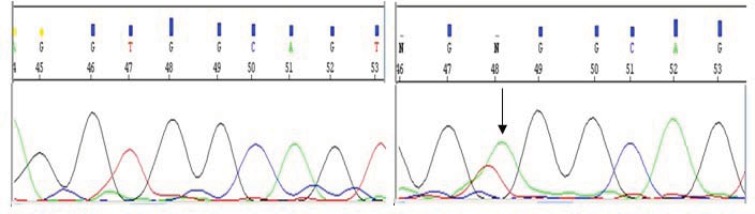
Result of gene sequensing in patient with W515R mutation in c-MPL gene, from right to left wild type and mutant allele

**Table I:** JAK2V617F mutation status in studied patients and its association with clinical and laboratory findings

**Characteristics**		**Jak2 V617F mutation**		
		positive	negative	P value	
**Subtype** **N (%)**	ET	31 (91.2)	15 (57.7)	0.02	
	PMF	3 (8.8)	11 (42.3)	0.02	

**Demographics** **N (%)**	Female	20 (58.8)	10 (38.5)	0.1	
	Male	14 (41.2)	16 (61.5)	0.1	
**Mean±SD** **(years old)**	Age	62.03±13.15	61.81±13.25	0.9	

**Laboratory test**	Hemoglobin (g/dL), mean (±SD)	13.18(±1.67)	11.58(±2.26)	0.003	
	White cells (*10^3^/µL) median (Q1-Q3)	16.03(9.0-20.5)	12.15(6.0-13.25)	0.005	
	Platelet count(*10^3^/µL) Median (Q1-Q3)	895.8(759.5-1062)	682.1(319.5-903.2)	0.03	

**Sympotom** **N (%)**	Splenomegaly	20 (58.5)	16 (61.5)	0.3	
	Splenectomy	1 (2.9)	3 (11.5)	0.3	
	Normal spleen	13 (38.2)	7 (26.9)	0.3	

**TableII:** Clinical and laboratory findings in only patient with mutation in c-MPL gene

**findings** **Treatment**	** WBC count** *10^3^**/µl**	**Hb(g/dl)**	**Platelet count** *10 ^3^ **/** **µ** **l**	**The type and dosage of medicine**	**Status of spleen**
**At the time of diagnosis **	8.4	14.4	897	-	splenomegaly
**Four months after treatment **	3.8	13	321	Hydroxyurea 500 mg Daily , ASA 80 mg daily	normal
**Six months after treatment**	6.43	14	598	Hydroxyurea 500 mg Daily , ASA 80 mg daily	normal

## Discussion

The activation of JAK/STAT pathway has an important role in pathogenesis of chronic myeloproliferative disorders. JAK2V617F mutation has been identified in 95% of PV cases and about 50% of PMF and ET [5]. Studies to find the etiology of JAK2V617F-negative PMF or ET disorders leading to the identification of mutation in c-MPL gene. c-MPL mutations has a role in pathogenesis of ET and PMF. High expression of c-MPL mutation in ex-vivo models leads to cell growth enhancement without any dependence to growth factors. Other studies have shown that expression of mutant MPLW515L in transplanted mice leads to a type of phenotype like as myelofibrosis [12, 13]. It is shown that patients with c-MPL gene mutation have also low hemoglobin concentration, high platelet count (ET patient), and the risk of disorder of small arteries[14].In the present study, from 60 patients, 34 (57%) were positive for JAK2V617F mutation and 1 patient (1.7%) was positive for c-MPL mutation. Akpınar et al. studied 77 patients with ET (66 patients) and PMF (11 patients) and showed that 35 patients were positive for the JAK2V617F mutation. In addition, MPL-515L/K mutation in 2 patients (2.6%) were negative for JAK2V617F mutation[15]. This study confirms our results in terms of Xu et al. also investigated Chinese patients to screen MPLW515L and JAK2V617F mutation and found the presence of MPLW515L mutation in only one patient of 102, who had ET (1%)[16]. This investigation has similar results as our study. Results of Pardanani’s study revealed that mutations were positive in 1% of ET patients and 5% of PMF patients.Our results about ET patients was In line with Pardanani ‘s findings , both of them were about 1% . In this study, Jak2V617F mutation were positive respectively in 59% and 33% of ET and PMF patients that was almost identical with our findings[8]. However, the result of study of Lieu and colleagues on 105 Taiwanese patients with myeloproliferative disorders revealed that all patients were negative for c-MPL gene mutations[17]. Karimzadeh et al. and Asghari et al. investigated the prevalence of Jak2V617F mutation in Iranian patients with chronic myeloproliferative disorders. Their investigations revealed that this mutation were positive in 45%, 53% of ET and 63% of PMF patients, respectively. Our study differed from aforementioned studies in terms of PMF patients. Our study also revealed that patients with Jak2V617F mutation had higher WBC count and hemoglobin concentration average than others without mutation (p=0.005, p=0.003). 

## Conclusion 

The results of current study indicated that c-MPL gene mutations unlike Jak2V617F mutation were low in Iranian MPD patients. Due to the low prevalence of these mutations, it is not economically cost effective to recommend this test for screening of all patients with MPD . However, due to special nature of the c-MPL gene mutations in MPD patients (unlike Jak2V617F mutation) that has seen just in ET and PMF subgroups, this test could be helpful for diagnosis of disease in suspected cases. 

## Conflict of interest

There is no conflict of interest associated with this study.
